# Partner hygiene–related behaviors and vaginitis subtypes: an exploratory observational study

**DOI:** 10.1590/1806-9282.20260806

**Published:** 2026-10-23

**Authors:** Duygu Ucar Kartal, Mehmet Emre Peker, Ufuk Atlihan, Murat Ozsahin, Haydar Kaya

**Affiliations:** 1 Manisa Merkezefendi State Hospital, Department of Obstetrics and Gynecology – Manisa, Turkey.; 2 Izmir Democracy University, Buca Seyfi Demirsoy Training and Research Hospital, Department of Obstetrics and Gynecology – İzmir, Turkey.; 3 Manisa City Hospital, Department of Obstetrics and Gynecology – Manisa, Turkey.

**Keywords:** Vaginitis, Hygiene, Vaginosis, bacterial, Candidiasis, vulvovaginal, Trichomonas vaginitis

## Abstract

**OBJECTIVE:**

The aim of this study was to investigate the association between partner hygiene behaviors, quantified using an exploratory partner hygiene score, and vaginitis subtypes, and to evaluate the relationship between baseline partner hygiene and short-term recurrence.

**METHODS:**

This prospective observational study included women aged 18–45 years presenting with vaginitis symptoms to a gynecology outpatient clinic. Vaginitis subtypes were diagnosed using standardized clinical and laboratory criteria. Partner hygiene behaviors were assessed using a seven-item partner hygiene score (range: 0–14). Participants were followed for 3 months to assess clinically confirmed symptomatic recurrence. Associations between partner hygiene score and vaginitis subtypes were analyzed cross-sectionally, while recurrence analyses were performed prospectively.

**RESULTS:**

A total of 108 women were included. Diagnoses included bacterial vaginosis (45.4%), vulvovaginal candidiasis (29.6%), *Trichomonas vaginalis* infection (10.2%), mixed vaginitis (11.1%), and non-specific vaginitis (3.7%). Median partner hygiene score scores were significantly lower in women with bacterial vaginosis and trichomoniasis compared with vulvovaginal candidiasis (p<0.001). Each one-point decrease in partner hygiene score was associated with higher odds of bacterial vaginosis. During follow-up, recurrence occurred in 33% of cases, but baseline partner hygiene score was not associated with recurrence.

**CONCLUSION:**

Lower partner hygiene scores were associated with dysbiosis-predominant vaginitis phenotypes, particularly bacterial vaginosis and trichomoniasis. Although no association was observed with short-term recurrence, these findings are hypothesis-generating and require confirmation in larger prospective studies.

## INTRODUCTION

Vaginal infections—particularly bacterial vaginosis (BV), vulvovaginal candidiasis, and *Trichomonas vaginalis* infection—represent a major clinical and public health concern worldwide, with BV affecting approximately 23–29% of women of reproductive age globally, vulvovaginal candidiasis affecting up to 75% of women during their lifetime, and *T. vaginalis* infection accounting for an estimated 156 million new cases annually^1,2,3,4^. Central to these conditions is disruption of the *Lactobacillus*-dominated vaginal ecosystem, which predisposes to dysbiosis, polymicrobial biofilm formation, and increased susceptibility to sexually transmitted infections and poor pregnancy outcomes^5^.

Despite extensive research on host-related determinants, emerging evidence indicates that vaginal infections cannot be fully understood without accounting for partner-level contributors. Penile microbiota composition, particularly anaerobe-enriched communities, has been strongly linked to incident BV in female partners, while male circumcision modifies this microbial ecology and may reduce BV and HIV risk^6,7^. Yet findings remain inconsistent, underscoring the complexity of partner influences^8^.

Partner-related behavioral and hygiene factors may influence vaginal infection susceptibility; however, these variables have rarely been assessed using a standardized quantitative framework^9-17^. The lack of a standardized assessment of partner hygiene remains an important gap in vaginitis research^14,17,18^.

Therefore, this study aimed to explore the cross-sectional relationship between partner hygiene–related behaviors and common vaginitis subtypes among symptomatic women attending a gynecology outpatient clinic. Secondary analyses explored associations between baseline partner hygiene and 3-month recurrence.

## METHODS

Women aged 18–45 years who presented with complaints of vaginal discharge, odor, and/or itching, reported a history of sexual intercourse within the preceding 3 months, and were clinically diagnosed with vaginitis were consecutively included in the study. Pregnant women, those who had used antibiotics within the previous 4 weeks, women with known immunodeficiency, and those without a sexual partner were excluded. Women older than 45 years and those with postmenopausal status were excluded to minimize the potential influence of menopausal hormonal changes on the vaginal microbiota were excluded to minimize the potential influence of perimenopausal hormonal changes on the vaginal microbiota. Women with known immunodeficiency were excluded because of their increased susceptibility to atypical and recurrent vaginal infections, which could confound the association between partner hygiene and vaginitis subtypes.

All participants provided written informed consent prior to enrollment. The study protocol was approved by the Ethics Committee of the Istanbul Esenyurt University (Approval No. 2025/10-21) and was conducted in accordance with the principles of the Declaration of Helsinki.

Participants completed a structured questionnaire covering sociodemographic characteristics, reproductive history, sexual practices, personal hygiene habits, and previous history of vaginal infections.

Clinical diagnosis of vaginitis was established by standardized gynecologic examination supported by laboratory findings, including vaginal pH assessment, microscopy, and culture where indicated. BV was diagnosed using the Amsel criteria (≥3 of 4 criteria) and/or a Nugent score of 7–10. Vulvovaginal candidiasis was diagnosed based on compatible clinical findings together with microscopy and/or positive fungal culture. Mixed vaginitis was defined as the simultaneous presence of diagnostic criteria for two or more vaginal infections. Non-specific vaginitis was defined as symptomatic vaginitis in the absence of diagnostic criteria for BV, vulvovaginal candidiasis, or *T. vaginalis* infection. All clinical examinations and diagnostic assessments were performed by a single experienced gynecologist according to predefined standardized diagnostic criteria. Therefore, inter-rater reliability was not applicable.

### Treatment

All participants received treatment according to current international guidelines and the diagnosed vaginitis subtype. Women with BV received oral metronidazole (500 mg twice daily for 7 days). Vulvovaginal candidiasis was treated with a single 150-mg oral dose of fluconazole or topical azole therapy, as clinically indicated. Patients with *T. vaginalis* infection received oral metronidazole (500 mg twice daily for 7 days), and partner treatment was recommended according to current guidelines. Mixed vaginitis was treated according to the identified pathogens.

### Partner hygiene score

Partner hygiene behaviors were quantitatively assessed using the partner hygiene score (PHS), a composite score consisting of seven equally weighted items evaluating cleansing frequency, cleansing product type, underwear change frequency, pre- and post-coital hygiene, genital hair management–related skin integrity, and recurrent hygiene-related genital irritation. The items were selected based on their biological plausibility and clinical relevance to partner hygiene practices potentially influencing the vaginal microenvironment. Each item was scored from 0 to 2, yielding a total score ranging from 0 to 14, with higher scores indicating better partner hygiene. The PHS items were developed based on clinically relevant partner hygiene behaviors considered potentially associated with vaginal health, and the rationale for item selection was based on current clinical knowledge and literature findings.

The total PHS score was calculated and analyzed as a continuous variable, with higher scores indicating better partner hygiene. The total PHS score ranged from 0 to 14 and was analyzed both as a continuous variable and by tertiles. Internal consistency of the seven-item PHS was assessed using Cronbach’s alpha, demonstrating acceptable internal consistency (Cronbach’s α=0.793). The complete PHS questionnaire, item descriptions, and scoring instructions are provided in Supplementary Appendix 1.

### Follow-up and outcome measures

Participants were followed prospectively for 3 months after initial diagnosis and treatment. Treatment adherence was assessed at the 3-month follow-up visit by patient self-report. Women who reported that they had not completed the prescribed treatment regimen (n=5) were excluded from the recurrence analysis. Consequently, recurrence analyses were performed in 103 women. At the 3-month follow-up visit, all participants underwent clinical reassessment, and recurrent vaginitis was diagnosed using the same predefined diagnostic criteria applied at baseline. Recurrence was defined as the reappearance of symptomatic vaginitis confirmed by clinical and laboratory evaluation during the follow-up period.

The primary outcome:

- Vaginitis subtype at baseline (BV, VVC, TV, mixed, non-specific).

Secondary outcome (exploratory):

- Symptomatic recurrence within 3 months after treatment, confirmed by clinical/laboratory evaluation.

As this was an exploratory observational study, no formal a priori sample size calculation was performed. Statistical analyses were performed using SPSS version 24.0. Continuous variables were presented as mean±standard deviation or median (interquartile range), as appropriate. Categorical variables were summarized as frequencies and percentages. Comparisons between groups were performed using Student’s t-test or Mann-Whitney U test for continuous variables and χ^2^ test or Fisher’s exact test, as appropriate, for categorical variables. Multivariable logistic regression models were constructed to assess the independent association between the PHS score and BV. To minimize the risk of model overfitting given the available sample size, variables were selected a priori based on established clinical relevance and evidence from previous literature. The final adjusted model included age, body mass index, smoking status, number of sexual partners during the previous 12 months, vaginal douching, and PHS score. The variable of having ≥2 sexual partners during the previous 12 months was retained in the multivariable model based on its established relevance as a potential behavioral confounder, despite not reaching statistical significance in univariable analysis. Adjusted odds ratios (aORs) with 95%CIs were calculated. A two-sided p<0.05 was considered statistically significant. Given the exploratory nature of the study, p-values were not adjusted for multiple comparisons and should be interpreted as exploratory. For categorical comparisons involving PHS tertiles, χ^2^ test results were interpreted cautiously because of small cell sizes in some subgroup comparisons. A post-hoc sensitivity analysis was performed using G*Power version 3.1 (Heinrich Heine University, Düsseldorf, Germany). For the recurrence analysis (n=103), the minimum detectable effect size was Cohen’s d=0.593 (α=0.05, power=80%).

## RESULTS

A total of 152 women were assessed for eligibility during the study period. After applying the inclusion and exclusion criteria, 131 participants were enrolled. A total of 23 participants were excluded from the final analysis due to loss to follow-up or incomplete questionnaires and clinical data, resulting in a final study population of 108 women. The baseline demographic and clinical characteristics of the study population are presented in Table 1. The mean age of the participants was 31.1±5.8 years, and all reported sexual intercourse within the preceding 3 months. The most common presenting complaints were vaginal discharge (76.5%), itching (62.7%), and malodor (51.0%).

**Table 1:** Baseline demographic, behavioral, and clinical characteristics of the study population (n=108).

Characteristic	Value
Demographic characteristics	
Age (years), mean±SD	31.1±5.8
Body mass index (kg/m^2^), mean±SD	26.1±4.3
Higher education*, n (%)	58 (53.7)
Middle/high socioeconomic status†, n (%)	66 (61.1)
Current smoking, n (%)	31 (28.7)
Alcohol use‡, n (%)	30 (27.8)
Reproductive and sexual characteristics	
Gravidity, median (IQR)	2 (1–3)
Parity, median (IQR)	1 (1–2)
Nulliparous, n (%)	32 (29.6)
Previous vaginal delivery, n (%)	58 (53.7)
Previous cesarean section, n (%)	39 (36.1)
History of abortion, n (%)	24 (22.2)
≥2 sexual partners during the previous 12 months, n (%)	13 (12.0)
Sexual intercourse frequency, times/week, median (IQR)	2 (1–3)
Vaginal douching (occasionally or regularly), n (%)	39 (36.1)
Contraceptive method, n (%)	
No contraception	33 (30.6)
Condom	21 (19.4)
Combined oral contraceptive	14 (13.0)
Intrauterine device	26 (24.1)
Withdrawal	11 (10.2)
Other	3 (2.8)
Presenting symptoms§, n (%)	
Vaginal discharge	83 (76.5)
Vulvovaginal itching	68 (62.7)
Malodor	55 (51.0)
Burning sensation	29 (26.9)
Dyspareunia	18 (16.7)
Dysuria	14 (13.0)

*Data are presented as mean±SD, median (IQR), or number (percentage), as appropriate. *Higher education=high school or university. †Middle/high socioeconomic status=middle or high income level. ‡Alcohol use=occasional or regular alcohol consumption. §Participants could report more than one symptom. Values for symptoms are presented as frequencies and percentages; percentages may not sum to 100% because participants could report more than one symptom. SD: standard deviation; IQR: interquartile range.

Based on clinical and laboratory evaluation, 45.4% (n=49) of participants were diagnosed with BV, 29.6% (n=32) with vulvovaginal candidiasis, 10.2% (n=11) with *T. vaginalis* infection, and 11.1% (n=12) with mixed vaginitis. The remaining 3.7% (n=4) were classified as non-specific vaginitis. The four women with non-specific vaginitis were reported descriptively only and were not included in the comparative analyses of vaginitis subtypes.

The median PHS for the overall study population was 7 (interquartile range [IQR]: 6–10), with scores ranging from 3 to 14. Median PHS scores were 6 (IQR: 6–8) in women with BV, 6 (IQR: 5.5–7) in those with trichomoniasis, and 10 (IQR: 8.75–11) in women with vulvovaginal candidiasis. Comparison of PHS scores across diagnostic groups demonstrated lower PHS values in BV and trichomoniasis compared with candidiasis (Kruskal-Wallis test, p<0.001). Figure 1 shows the distribution of PHS scores according to vaginitis subtype.

**Figure 1:**
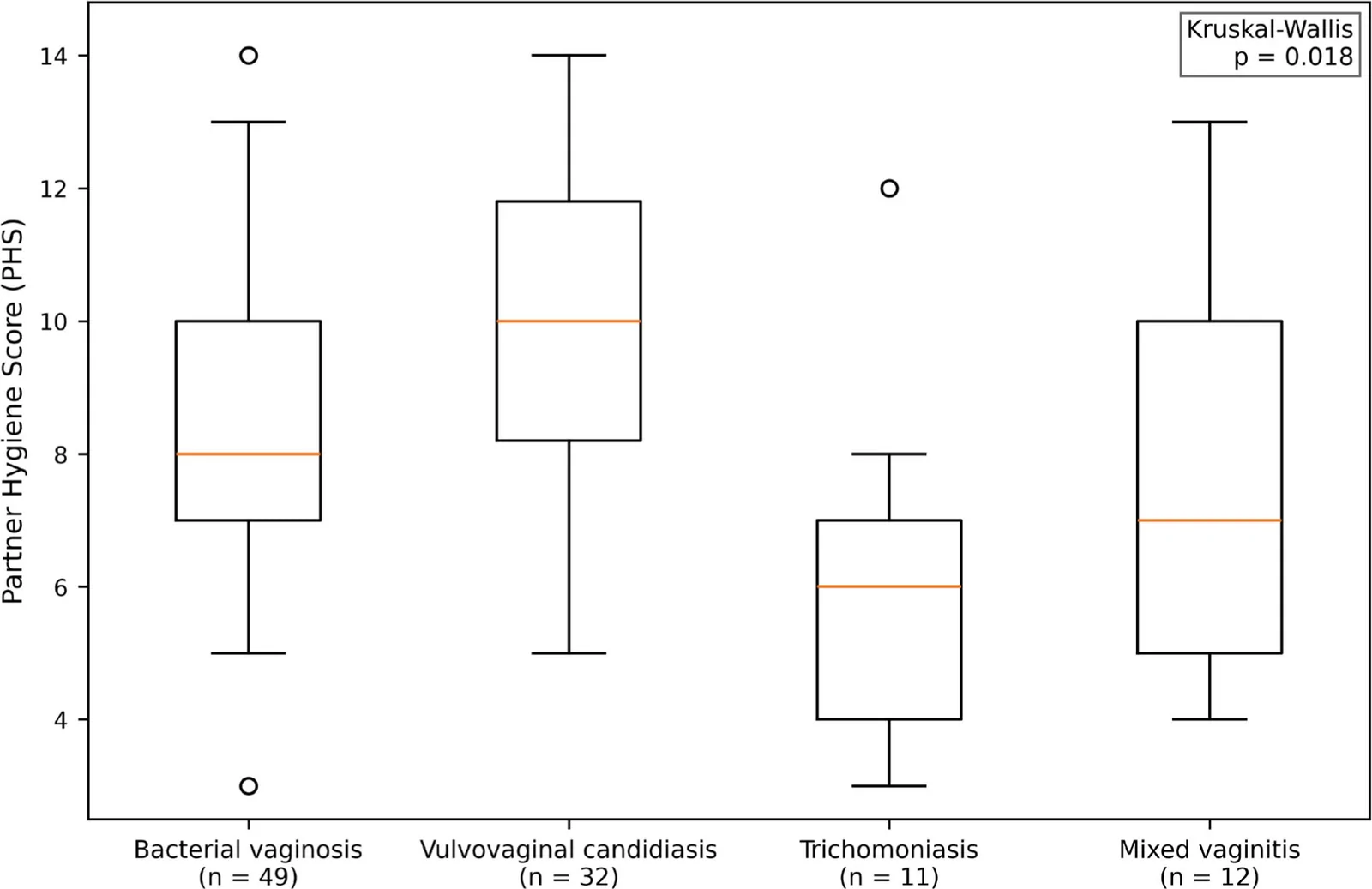
Distribution of partner hygiene score scores across vaginitis subtypes. Boxes represent the interquartile range, the horizontal line indicates the median, whiskers represent values within 1.5 × interquartile range, and circles denote outlier observations. Sample sizes are shown on the x-axis. Differences among groups were assessed using the Kruskal-Wallis test (p<0.001).

In the analysis stratified by PHS tertiles, the prevalence of BV (55.1%) and trichomoniasis (54.5%) was higher in the lowest PHS tertile compared with the middle and highest tertiles (p<0.001). In contrast, vulvovaginal candidiasis was more frequently observed in the highest PHS tertile. The distribution of PHS scores and PHS tertiles according to vaginitis subtype is summarized in Table 2.

**Table 2:** Partner hygiene score scores and tertile distribution according to vaginitis subtype.

Vaginitis subtype	n	PHS median (IQR)*	PHS range	Low PHS tertile n (%)	Middle PHS tertile n (%)	High PHS tertile n (%)
Bacterial vaginosis	49	6 (6–8)	3–14	27 (55.1)	14 (28.6)	8 (16.3)
Vulvovaginal candidiasis	32	10 (8.75–11)	5–14	6 (18.8)	9 (28.1)	17 (53.1)
*Trichomonas vaginalis*	11	6 (5.5–7)	3–10	6 (54.5)	4 (36.4)	1 (9.1)
Mixed vaginitis	12	8.5 (6.75–10.25)	4–13	(25.0)	5 (41.7)	4 (33.3)

*Statistical analysis: PHS scores: Kruskal-Wallis test (χ^2^=19.89, p<0.001). PHS tertile distribution: Fisher-Freeman-Halton exact test, p=0.003. Fisher-Freeman-Halton exact test was used because some expected cell counts were <5. PHS: partner hygiene score; IQR: interquartile range. PHS tertile cut-points were defined as follows: Low tertile, 3–6; Middle tertile, 7–9; High tertile, 10–14.

In univariable logistic regression analysis, each one-point increase in the PHS score was associated with a lower likelihood of BV (OR 0.79, 95%CI 0.67–0.94, p=0.007). Current smoking (OR 3.01, 95%CI 1.26–7.17, p=0.013) and vaginal douching (OR 3.35, 95%CI 1.47–7.61, p=0.004) were also significantly associated with BV in the univariable analysis. The results of the univariable and multivariable logistic regression analyses are presented in Table 3. In the multivariable logistic regression analysis, higher PHS scores remained independently associated with lower odds of BV after adjustment for age, body mass index, smoking status, number of sexual partners during the previous 12 months, and vaginal douching (adjusted OR 0.80, 95%CI 0.67–0.96, p=0.019). Current smoking (adjusted OR 2.90, 95%CI 1.12–7.51, p=0.028) and vaginal douching (adjusted OR 2.91, 95%CI 1.20–7.03, p=0.018) were also independently associated with BV. Body mass index, age, and having two or more sexual partners during the previous 12 months were not independently associated with BV.

**Table 3:** Univariable and multivariable logistic regression analyses for factors associated with bacterial vaginosis.

Variable	Univariable OR (95%CI)	p	Adjusted OR (95%CI)	p-value
PHS score (per 1-point increase)	**0.79 (0.67–0.94)**	**0.007**	**0.80 (0.67–0.96)**	**0.019**
Age (per year)	0.97 (0.91–1.03)	0.340	0.95 (0.88–1.03)	0.199
Body mass index (per kg/m^2^)	0.95 (0.87–1.04)	0.282	0.98 (0.88–1.09)	0.751
Current smoking	**3.01 (1.26–7.17)**	**0.013**	**2.90 (1.12–7.51)**	**0.028**
≥2 sexual partners (previous 12 months)	1.47 (0.46–4.71)	0.515	1.02 (0.27–3.87)	0.971
Vaginal douching (yes)	**3.35 (1.47–7.61)**	**0.004**	**2.91 (1.20–7.03)**	**0.018**

OR: odds ratio; CI: confidence interval; PHS: partner hygiene score. Bold values indicate statistically significant associations (p<0.05); the corresponding ORs and 95%CIs are also bolded for emphasis.

When PHS components were evaluated individually, non-daily partner underwear changing was more frequently reported among women with BV and demonstrated a borderline statistical association (p≈0.08). No statistically significant associations were observed between other PHS components and vaginitis types.

During the 3-month follow-up period, vaginitis recurrence was observed in 33% of cases (n=34). The recurrence rate was higher among women initially diagnosed with BV, followed by mixed vaginitis and *T. vaginalis* infection, while it was lower among those diagnosed with vulvovaginal candidiasis. No statistically significant association was found between overall PHS scores and vaginitis recurrence during the 3-month follow-up period.

## DISCUSSION

In this exploratory clinical study, lower partner hygiene—as quantified by a newly developed PHS—was associated with a dysbiosis-predominant vaginitis phenotype. Specifically, PHS scores were significantly lower in women diagnosed with BV and *T. vaginalis* infection compared with VVC, and lower PHS tertiles were enriched for BV and trichomoniasis. In regression analyses, each one-point decrease in PHS was associated with higher odds of BV after adjustment for potential confounders. By contrast, PHS was not associated with 3-month recurrence, despite an overall recurrence rate of approximately one-third.

Although the association between PHS and BV remained statistically significant after multivariable adjustment (adjusted OR 0.80, 95%CI 0.67–0.96), the observed effect size was modest. Therefore, these findings should be interpreted cautiously and require confirmation in larger prospective studies before any clinical implications can be drawn. Although vaginal douching and smoking showed similar effect estimates in the regression model, these factors may partially overlap with broader hygiene-related behaviors and lifestyle patterns. However, vaginal douching and smoking were included as covariates in the multivariable model to minimize potential confounding of the association between PHS and BV.

The recurrence analysis should also be interpreted cautiously, as only 34 recurrence events were observed during follow-up. Consequently, the study may have been underpowered to detect modest associations between baseline PHS and recurrence, and the absence of a statistically significant association should not be interpreted as evidence of no association. Larger prospective studies are warranted to further investigate this relationship.

Our findings support the concept that BV is linked to dysbiosis and partner-associated microbial exchange rather than exclusively host-related susceptibility^5,12,18^. Recent studies using culture-independent molecular techniques have substantially advanced our understanding of the relationship between the vaginal and penile microbiomes. Using 16S rRNA sequencing, Mehta et al.^6^ demonstrated that the composition of the penile microbiome predicts incident BV in female partners. Consistent with these findings, concurrent treatment of male partners has been shown to alter the genital microbiota of couples and may improve treatment outcomes, highlighting the importance of partner-associated microbial reservoirs^19^. Furthermore, prospective cohort studies have shown that sexual activity contributes to the persistence of a non-optimal vaginal microbiota following BV treatment^20^, while whole metagenome sequencing has provided direct evidence of microbial exchange between sexual partners^15^. These findings provide a biologically plausible explanation for the observed association between poor partner hygiene and BV, suggesting that poor partner hygiene may contribute to repeated microbial transfer and disruption of the vaginal microbiota. The hypothesized biological pathway linking these mechanisms is summarized in Figure 2. This conceptual model is based on current evidence and is intended to illustrate biological plausibility rather than establish causal relationships.

**Figure 2:**
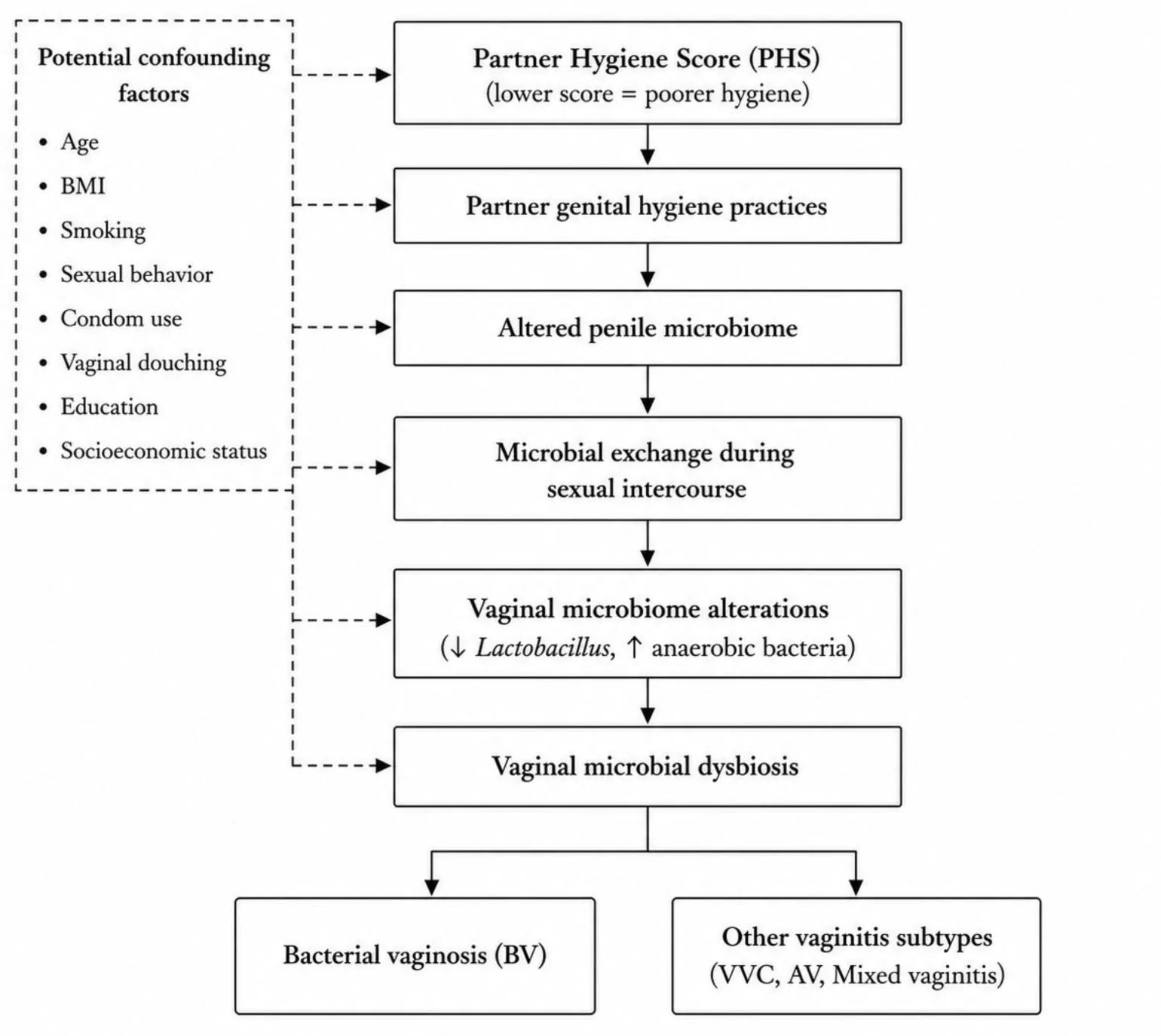
Proposed conceptual model linking partner hygiene to vaginitis subtypes. Solid arrows indicate the hypothesized biological pathway based on current evidence, whereas dashed arrows represent potential confounding factors considered in the statistical analyses. The model is intended to illustrate biological plausibility rather than establish causal relationships.

The particularly low PHS scores observed among women with trichomoniasis may be explained by the sexually transmitted nature of this infection and its established links with vaginal dysbiosis^21-23^. In contrast, VVC is more strongly associated with host-related and metabolic factors influencing the vaginal environment and microbiome composition than partner-mediated bacterial exchange^16,24^. The higher PHS scores among women with VVC in our cohort may therefore reflect a genuine subtype distinction: PHS may be more sensitive to behaviors that facilitate anaerobe-associated dysbiosis and sexually transmitted pathogens than to fungal overgrowth.

Among individual PHS components, non-daily partner underwear changing showed a borderline association with BV. Although not definitive, this is consistent with the hypothesis that specific, easily modifiable behaviors may be associated with microbial persistence or re-seeding between partners. The broader literature suggests that women frequently attribute BV onset and recurrence to sexual behaviors and partner factors, underscoring the clinical relevance of actionable, couple-focused counseling^25^. However, most prior work has evaluated single partner variables (e.g., circumcision status, smoking) rather than aggregating daily hygiene practices into a quantitative measure, limiting comparability across studies^12,13^. Our PHS framework addresses this gap by operationalizing partner hygiene as a multidimensional exposure, enabling modeling of dose–response patterns and subgroup analyses.

Despite evidence that BV is characterized by high recurrence and chronicity in many populations, we did not observe a significant relationship between baseline PHS and recurrence at 3 months^14,18,26^. The lack of association between baseline PHS and recurrence may reflect multifactorial recurrence mechanisms, treatment-related factors, and unmeasured behavioral changes during follow-up^5,9,12,27^.

Notably, partner-directed strategies for BV have shown renewed promise, including concurrent male partner treatment and combined antimicrobial approaches that alter couples’ genital microbiota^28^, although the broader evidence base remains mixed and continues to evolve^27^. Our null recurrence finding should therefore be interpreted cautiously and viewed as hypothesis-refining rather than hypothesis-negating. Beyond partner-directed strategies, emerging evidence suggests that oral probiotics may represent a complementary, partner-independent approach to supporting vaginal health and modulating the vaginal microbiome, particularly in specific populations such as postmenopausal women^24^. However, the role of probiotics in preventing recurrence in premenopausal women with BV remains to be established and warrants investigation alongside partner hygiene interventions.

Clinically, these findings support a more partner-inclusive approach to vaginitis phenotyping—particularly for BV and trichomoniasis—by recognizing that partner behaviors may be associated with exposure to dysbiosis-associated bacterial communities and sexually transmitted pathogens^15,18,23^. While routine “partner hygiene scoring” is premature, the PHS concept could be used to structure targeted counseling on feasible behaviors (e.g., regular underwear changing and consistent genital hygiene around intercourse), alongside evidence-based STI management and prevention.

Although the theoretical PHS range was 0–14, the observed scores in our study ranged from 3 to 14. The absence of scores at the extreme lower end may indicate limited representation of very poor partner hygiene behaviors in this sample; however, formal assessment of floor or ceiling effects was not performed. For research, PHS requires formal validation (reliability, construct validity, and responsiveness) and should be tested in larger, multi-center cohorts with repeated measurement across time. Test–retest reliability of the PHS should be evaluated in future validation studies. Integration of penile and vaginal microbiome profiling would be particularly valuable to determine whether PHS correlates with anaerobe-enriched penile communities or with strain-sharing within couples, strengthening causal inference^6,28^. Finally, intervention studies could examine whether improving PHS-targeted behaviors—alone or combined with partner treatment—are associated with lower BV incidence or recurrence and whether effects differ by baseline subtype and biofilm burden^5,19^.

Key strengths include the introduction of a novel, quantitative partner hygiene measure and the subtype-stratified analysis across common vaginitis diagnoses. Limitations include reliance on participant-reported partner behaviors (potential misclassification and social desirability bias), the single-center design, which may limit the generalizability of the findings to other populations and healthcare settings, residual confounding by female intravaginal practices and sexual network factors, and the relatively small sample size, particularly in the trichomoniasis and other smaller diagnostic subgroups, which may have limited the statistical power and precision of subgroup analyses. In addition, partner treatment status, changes in partner hygiene during follow-up, exact time to recurrence, and other time-varying factors potentially associated with recurrence were not prospectively recorded. Therefore, time-to-event analyses such as Cox proportional hazards regression could not be performed, and the recurrence analysis should be interpreted as exploratory. Additionally, the PHS is a newly developed exploratory instrument and has not undergone formal psychometric validation; therefore, its reliability and validity should be confirmed in future studies. Moreover, objective validation of the PHS against external measures, such as partner interviews or microbiological assessments, was not performed in this study. Since p-values were not adjusted for multiple comparisons, significant findings should be interpreted as exploratory.

## CONCLUSION

Overall, poorer partner hygiene as captured by PHS was associated with BV and trichomoniasis compared with VVC. These findings indicate subtype-specific cross-sectional associations between partner hygiene and vaginitis phenotypes. The absence of a clear association with 3-month recurrence indicates that recurrence is likely driven by additional factors beyond baseline partner hygiene alone. Given the exploratory nature of the recurrence analysis, these findings should be considered hypothesis-generating and require prospective validation in larger cohorts. Furthermore, before clinical implementation, the PHS requires prospective validation and formal psychometric evaluation to establish its reliability, validity, and clinical applicability.

## Data Availability

The original datasets used in this study are publicly available from their respective sources. The merged analytical dataset, including derived variables created for the analyses, is available from the corresponding author upon reasonable request.
